# Exploring Non-Metabolic Functions of Glycolytic Enzymes in Immunity

**DOI:** 10.3389/fimmu.2017.01549

**Published:** 2017-11-22

**Authors:** Scott M. Seki, Alban Gaultier

**Affiliations:** ^1^Center for Brain Immunology and Glia, Department of Neuroscience, Charlottesville, VA, United States; ^2^Graduate Program in Neuroscience, Charlottesville, VA, United States; ^3^Medical Scientist Training Program, Charlottesville, VA, United States

**Keywords:** immunometabolism, inflammation, glyceraldehyde-3-phosphate dehydrogenase, hexokinase, pyruvate kinase, lactate dehydrogenase, glycolysis

## Abstract

At the beginning of the twentieth century, discoveries in cancer research began to elucidate the idiosyncratic metabolic proclivities of tumor cells (1). Investigators postulated that revealing the distinct nutritional requirements of cells with unchecked growth potential would reveal targetable metabolic vulnerabilities by which their survival could be selectively curtailed. Soon thereafter, researchers in the field of immunology began drawing parallels between the metabolic characteristics of highly proliferative cancer cells and those of immune cells that respond to perceived threats to host physiology by invading tissues, clonally expanding, and generating vast amounts of pro-inflammatory effector molecules to provide the host with protection. Throughout the past decade, increasing effort has gone into elucidating the biosynthetic and bioenergetic requirements of immune cells during inflammatory responses. It is now well established that, like tumor cells, immune cells must undergo metabolic adaptations to fulfill their effector functions (2, 3). Unraveling the metabolic adaptations that license inflammatory immune responses may lead to the development of novel classes of therapeutics for pathologies with prominent inflammatory components (e.g., autoimmunity). However, the translational potential of discoveries made toward this end is currently limited by the ubiquitous nature of the “pathologic” process being targeted: metabolism. Recent works have started to unravel unexpected non-metabolic functions for metabolic enzymes in the context of inflammation, including signaling and gene regulation. One way information gained through the study of immunometabolism may be leveraged for therapeutic benefit is by exploiting these non-canonical features of metabolic machinery, modulating their contribution to the immune response without impacting their basal metabolic functions. The focus of this review is to discuss the metabolically independent functions of glycolytic enzymes and how these could impact T cells, agents of the immune system that are commonly considered as orchestrators of auto-inflammatory processes.

## Introduction

Upon activation, T cells increase biomass, proliferate, and produce inflammatory cytokines—processes that are bioenergetically and biosynthetically demanding, and likewise, necessitate a conversion from a relatively quiescent metabolism (2–5). One mechanism by which this is accomplished is through elevated glycolytic flux. As a result, many groups are pursuing the promise of anti-glycolytic therapy for inflammatory indications (6, 7). Conversely, there is also interest in interventions to restore T cell metabolism in diseases of pathologic immunosuppression (e.g., cancer) (8–10). Intriguingly, many glycolytic enzymes serve moonlighting functions in the cell that can impact the nature and quality of an inflammatory response. Such idiosyncrasies may represent exploitable opportunities by which immune responses may be therapeutically modulated. The goal of this review is to present non-metabolic functions of glycolysis enzymes and the ways in which these idiosyncrasies may be exploited to impact inflammatory responses, particularly those of T cells.

## Glycolysis Enzymes and Their Roles in Inflammation

### Hexokinase II (HK-II)

**Figure 1:**
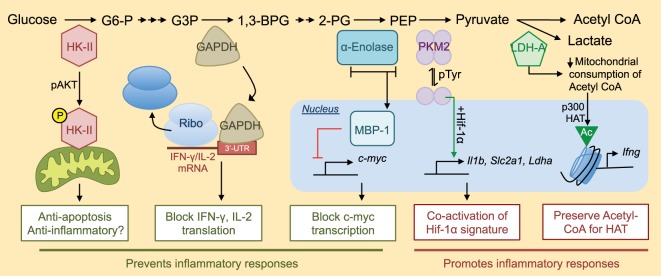
Non-metabolic functions of glycolytic enzymes and their roles in inflammation. Many pieces of glycolytic machinery have non-metabolic functions that can contribute to the inflammatory response. An abridged version of the glycolytic cascade is listed with enzymes depicted at their appropriate level in glycolysis along with their alternative non-metabolic functions. For a more complete view of the glycolytic cascade, please see Ref. (17). G6-P, glucose 6-phosphate; G3P, glyceraldehyde 3-phosphate; 1,3-BPG, 1,3-bisphosphoglycerate; 2-PG, 2-phosphoglycerate; PEP, phosphoenolpyruvate; Ribo, ribosome; Slc2a1, gene encoding glucose transporter 1 (Glut-1); HAT, histone acetyltransferase.

Hexokinase is the first enzyme involved in glycolysis, catalyzing the phosphorylation of glucose to glucose 6-phosphate (G6P) (Figure 1). Induction of HK-II, one of four isoforms of hexokinase, appears to be tightly linked to activation of inflammatory programs in immune cells (11, 12) and tumorigenic programs in cancer cells (10). Phosphorylated AKT stabilizes the localization of HK-II to the outer mitochondrial membrane (OMM). At this location, mitoHK-II has increased access to mitochondrially derived ATP, which it can then use to phosphorylate glucose to G6P, thereby trapping glucose in the cell (13). MitoHK-II also plays an anti-apoptotic role, preventing the formation of the mitochondria permeability transition pore by Bcl-2 family proteins like Bax (14, 15). The mechanism behind this process involves PI3K-AKT-mediated phosphorylation of Thr473 in HK-II, a modification that prevents G6P-mediated dissociation of HK-II from the mitochondria (16). Thus, posttranslational modifications to HK-II both facilitate its activity as a glycolytic enzyme and promote its anti-apoptotic functions.

Upon activation, immune cells upregulate HK-II (17) as well as other HK family members (18). HK-targeted interventions block glycolysis, effector function, and survival of cells involved in driving inflammatory responses (6), and for myeloid cells, this is especially true in the context of gram-negative bacterial challenges (19). However, this may not be true of all inflammatory responses. *N*-acetylglucosamine, a peptidoglycan derivative from the cell wall of Gram-positive bacteria, has recently been shown to bind HK-II and promote its dissociation from the OMM. This dissociation results in the accumulation of mitochondrial DNA in the cytosol and NLRP3 inflammasome-dependent production of mature IL-1β and IL-18 in macrophages (20). Thus, while dissociation of HK-II from the OMM might, on the one hand, abrogate the efficiency of flux through the glycolytic cascade and thus block inflammation, on the other hand, it may potentiate signals that promote secretion of major soluble transducers of inflammation depending on context. Inflammasome components (21, 22), hexokinase (6), and mitochondrial dynamics (23) are all known modulators of T cell functions; however, whether or not HK relocalization can induce inflammasome activity in T cells, and what consequences this may have, remains unclear.

### Glyceraldehyde 3-Phosphate Dehydrogenase (GAPDH)

Glyceraldehyde 3-phosphate dehydrogenase is the enzyme that catalyzes conversion of glyceraldehyde 3-phosphate to 1,3-bisphosphoglycerate in glycolysis (Figure 1). GAPDH is well known for its numerous non-metabolic functions. In many bacteria, GAPDH is a major component of the cell surface. Multiple mechanisms are involved in this localization of GAPDH, including active transport (24) and lysis-mediated release of GAPDH which then decorates the surface of neighboring bacterial cells (25). Cell surface GAPDH binds fibronectin, plasminogen, and other tissue components (24–26) and is an important facilitator of bacterial adherence to and invasion of host tissues. These findings translate to eukaryotic systems. In response to inflammatory cues, macrophages recruit GAPDH to the cell surface where it functions as a plasminogen receptor. In this paradigm, plasminogen bound to GAPDH digests extracellular matrix thereby facilitating macrophage migration (27). GAPDH can also localize to numerous other subcellular compartments (28). For example, oxidative stress, as occurs during neutrophil respiratory burst, drives *S-*nitrosylation of GAPDH (29), redistributing it from the cytoplasm to the nucleus and mitochondria where it is broadly implicated as a regulator of cell survival [reviewed in Ref. (28)]. GAPDH itself has been shown to have anti-inflammatory properties, as systemic administration of GAPDH prior to LPS-induced sepsis reduces cytokine storm and mortality (30), though the mechanism of this immunomodulatory effect remains unknown.

Recent work in T cells implicates GAPDH as an energy sensor that regulates translation of inflammatory cytokine mRNA in response to the availability of glucose in the cell. When glucose concentrations are low, GAPDH binds to the AU-rich elements in the 3′-untranslated region (UTR) of mRNA, including those encoding interferon gamma (IFN-γ) and IL-2 (31, 32). Binding of GAPDH to these transcripts represses their translation, thus restricting cytokine production during glucose deprivation. 3′AU-rich elements are not unique features of IFN-γ and IL-2 mRNA, and it is likely that GAPDH can regulate translation beyond these two cytokines (33). The glycolytic reaction catalyzed by GAPDH requires nicotinamide adenine dinucleotide (NAD^+^), an essential indicator of cellular redox state, and intriguingly, Nagy and colleagues identified the NAD^+^ binding fold of GAPDH as its RNA-binding domain (34). This finding suggests any NAD^+^-dependent enzyme [in glycolysis, this is GAPDH and lactate dehydrogenase (LDH)] may be endowed with RNA-binding capabilities. Glucose deprivation, however, increases levels of intracellular NAD^+^ which might be expected to compete with GAPDH for RNA binding (35). Thus, there are likely additional layers of regulation governing the role of GAPDH as a translational repressor that functions during glucose deprivation and or in response to fluctuations in NAD^+^. Context-specific nuances that influence how NAD^+^ affects the mRNA-binding functions of glycolytic machinery offer an intriguing line of inquiry into the interplay between metabolism and the many fundamental processes (36, 37) regulated by NAD^+^.

### α-Enolase

α-Enolase catalyzes the conversion of 2-phosphoglycerate to phosphoenolpyruvate (PEP) in glycolysis (Figure 1). The gene that encodes α-enolase (*Eno1*) produces a single transcript with two translational start sites. Depending on the site of translation initiation, *Eno1* can generate a full-length canonical α-enolase (48 kDa) enzyme that participates in glycolysis, or a truncated version of α-enolase (37 kDa), also known as Myc promoter-binding protein 1 (MBP-1) that represses the pro-proliferative transcription factor c-myc (38–41). Wang and colleagues identified c-myc as the master regulator of metabolic adaptation in T cells (17), demonstrating impaired growth and proliferation in c-myc deficient T cells treated with mitogenic stimuli. MBP-1 represses c-myc by binding to and inhibiting formation of the transcription initiation complex at the c-myc promoter (40, 41). Whereas α-enolase localizes to the cytoplasm, MBP-1 preferentially traffics to the nucleus where it serves these repressive functions (38). The signals that influence differential translation of α-enolase versus MBP-1 are unclear, though hypoxia may be one cue that favors translation of full-length α-enolase (42). The internal translation start site that generates MBP-1 off of *Eno1* is not present in β or γ-enolase, potentially providing an added layer of specificity for future MBP-1 modulating interventions.

Intriguingly, it seems that the induction of MBP-1 functionally impacts T cell inflammatory responses in the context of autoimmunity. A recent study (43) revealed that an anti-inflammatory population of human CD4^+^ T cells, known as regulatory T cells (Tregs), expresses high levels of MBP-1. Moreover, MBP-1 in Tregs potentiates transcription of a specific spliced isoform of FoxP3 known to potently suppress inflammatory immune responses, particularly those mediated by the transcription factor RAR-related orphan receptor gamma T (RORγT). RORγT is a known driver of IL-17A (44) and granulocyte macrophage colony stimulating factor (GM-CSF) (45), pro-inflammatory cytokines strongly associated with auto-inflammatory diseases (46–48), and the therapeutic potential of its inhibition is under investigation for numerous inflammatory indications (49, 50). Interestingly, Tregs seem to elevate expression of both *Eno1* gene products, suggesting that the suppressive effects of MBP-1 may dominate over metabolic contributions to inflammation facilitated by full-length α-enolase or elevated glycolysis (43, 51). Thus, inducing transcriptional activity at *Eno1* may be sufficient to increase MBP-1 protein levels to immunosuppressive levels without blocking glycolysis. How the α-enolase/MBP-1 axis affects conventional T cell responses is unclear. Taken together, whereas *Hk2* encodes a single protein that can play metabolic and non-metabolic roles in a cell, *Eno1* encodes two gene products that differ drastically in their contributions to metabolism and inflammation (38, 39).

### Pyruvate Kinase (PK) Isoform M2

Pyruvate kinase is the ATP-generating enzyme that catalyzes the conversion of PEP to pyruvate during glycolysis (Figure 1). Four isoforms of the PK enzyme exist, with the M1 (PKM1) and M2 (PKM2) isoforms being most predominant in leukocytes of the adult animal (52). PKM2 is the major isoform expressed at the protein level by lymphocytes (52). Interestingly, many cancer cell lines also exclusively express PKM2 (53), and cancer researchers have likewise identified many pro-proliferative and non-canonical functions that are specifically attributed to this particular isozyme (54–63). PKM1 and PKM2 are alternatively spliced isoforms of the PK enzyme that differ by inclusion of a single exon (exon 9 for PKM1 versus exon 10 for PKM2), of which only 22 amino acid residues differ (64). The structures of PKM1 and PKM2 are extremely similar (65), but importantly, the minute difference in amino acid sequence allows PKM2 to uniquely contribute to proliferative responses in cancer cells and inflammatory responses of immune cells (66–69). Whereas PKM1 exists solely as a tetramer that functions as a glycolytic enzyme, PKM2 can exist as a tetramer with similar functions as PKM1 or as a dimer that loses activity as a glycolytic enzyme, but can perform numerous other non-glycolytic functions in the cell. From the perspective of glycolysis, this dynamic feature of PKM2 reduces its efficiency as a glycolytic enzyme and allows for the accumulation of upstream glycolytic intermediates, thereby promoting *de novo* amino acid and lipid biosynthesis—processes that are critical for the production of a daughter cell (70). From the perspective of inflammation, the PKM2 dimer can localize to the nucleus (58) where it is a well-known co-activator of Hif-1α gene signatures (54, 66, 67). In macrophages, this interaction is critical for the appropriate transcriptional activation of metabolic machinery, such as lactate dehydrogenase A (LDH-A) and pro-inflammatory cytokines, such as IL-1β (66). Similarly, signal transducer and activator of transcription 3 (STAT3) (55) and the aryl hydrocarbon receptor (AhR) (71) also require interaction with PKM2 for appropriate DNA binding. Thus, the PKM2 dimer seems to play a unique role as a direct modulator of proliferative and inflammatory programs. Relating to T cells, AhR, STAT3, and Hif-1α are all well-known regulators of Th17 cell differentiation perhaps implicating PKM2 as a regulator of this cell type.

Many groups in cancer research (56, 57, 60) and immunology (66–69, 72) are exploring the therapeutic potential of enforcing PKM2 tetramerization with pharmacologic compounds (62, 73). The major endogenous driver of PKM2 tetramerization is fructose 1,6 bisphosphate (FBP) (65), the product of the phosphofructokinase-catalyzed step in glycolysis. Phosphotyrosine residues generated by growth factor signaling (57, 59) can bind to PKM2 and promote release of FBP, and along with posttranslational modifications, such as PKM2 phosphorylation (74), oxidation (61), acetylation (58), and succinylation (75, 76), are endogenous drivers of tetramer dissociation. Synthetic activators of PKM2 tetramerization, originally characterized in cancer models as tumor-blocking agents (62), also potently block inflammation in numerous disease models (66, 67, 77). Thus, enforcing PKM2 tetramerization shows promise as a metabolic machinery-based paradigm for controlling inflammatory responses without overtly inhibiting metabolism itself.

### Lactate Dehydrogenase A

Lactate dehydrogenase is a tetrameric enzyme variably composed of A and B subunits that, when combined, form a complex with the capability of converting pyruvate to lactate (Figure 1). This reaction is the defining step of aerobic glycolysis (78), the form of metabolism engaged by activated immune cells, which increase their regeneration of NAD^+^ consumed during glycolysis by producing lactate regardless of environmental oxygen content (2, 3). Peng and colleagues (79) recently showed that T cells almost exclusively express the A subunits of LDH, which they further upregulate upon activation, and expression of LDH-A is critical for the proper production of the inflammation-promoting cytokine IFN-γ. They found that genetic ablation of LDH-A in T cells heightened consumption of glycolysis-derived acetyl-CoA through the tricarboxylic cylic acid cycle and depleting intracellular stores of this metabolic byproduct of glucose catabolism. This acetyl-CoA depletion impaired activation-induced permissive histone acetylations that are required for opening of the *Ifng* locus during T cell activation. These findings and others (80–82) suggest that metabolic adaptations like aerobic glycolysis are important (1) as a means of generating sufficient ATP and metabolic intermediates to support anabolic processes and (2) as drivers of the epigenetic changes that are responsible for facilitating engagement of the inflammatory program [reviewed in Ref. (83)]. In addition to its ability to indirectly modulate the epigenetic landscape of the activated T cell, there is evidence to suggest that LDH-A may also be capable of directly influencing inflammatory responses. In a manner reminiscent of direct repression of IFN-γ and IL-2 mRNA translation by the glycolytic enzyme GAPDH (31, 32), LDH-A has been reported to bind to 3′AU-rich elements in GM-CSF mRNA (84). It remains unclear how the mRNA-binding properties of LDH-A affects downstream protein expression and, additionally, if this non-metabolic function is related to the level of flux through the glycolytic cascade or enzymatic activity. Inflammatory T cells are major producers of GM-CSF, a prominent driver of autoimmune responses (45–47, 85, 86), and a detailed study elucidating the metabolic requirements for GM-CSF production *in vivo*, including how it may relate to LDH-A, is warranted.

## The Relationship Between Glycolysis and Inflammation *In Vivo*

**Figure 2:**
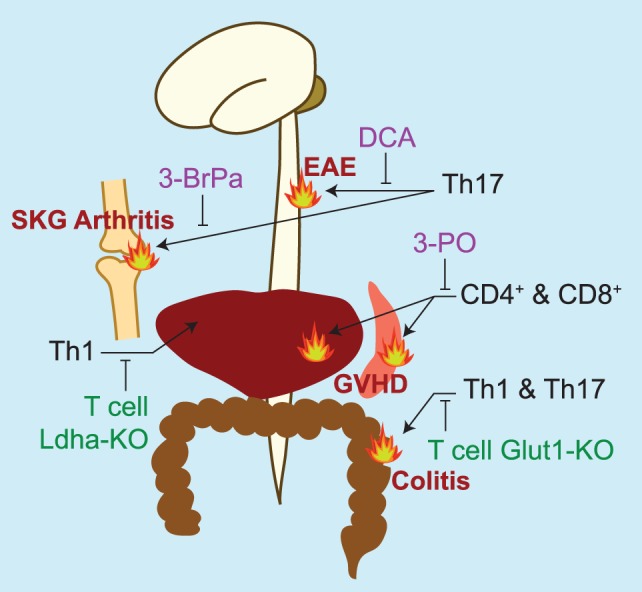
Summary of studies targeting glycolytic machinery *in vivo* to treat pathologies with prominent inflammatory T cell contributions. Pharmacologic inhibitors of glycolysis are listed in purple. DCA, dichloroacetate, an inhibitor of pyruvate dehydrogenase kinase 1 (12); 3-BrPa, 3-bromopyruvate, an inhibitor of hexokinase and GAPDH (93); 3-PO, 3-(3-pyridinyl)-1-(4-pyridinyl)-2-propen-1-one, an inhibitor of 6-phosphofructo-2-kinase/fructose-2,6-bisphosphatase 3 (PFKFB3, PFK2) (91). Genetic models targeting glycolytic machinery in T cells are listed in green. LDH-A, lactate dehydrogenase A (79); Glut-1, glucose transporter 1 (90). *In vivo* models of inflammation studied are experimental autoimmune encephalomyelitis (EAE)—a murine model of multiple sclerosis, SKG arthritis [a model of rheumatoid arthritis that spontaneously develops in the SKG strain of mice (99)]; graft versus host disease (GVHD), and colitis.

Seminal *in vitro* studies defined the metabolic peculiarities of inflammatory T cell subtypes (87–89) and paved the way for future works assessing the impact of glycolytic manipulations on T cell-driven inflammation *in vivo* (Figure 2) (10, 12, 90–94). Recent studies, however, question the strength of the relationship between glycolysis and inflammation in the *in vivo* setting. Peripheral blood T cells isolated from patients with rheumatoid arthritis show defects in glycolytic flux, rather than elevated glycolysis (95, 96). Likewise, impaired glycolysis is also detected in peripheral blood T cells isolated from multiple sclerosis patients and type 1 diabetics (43). One potential explanation for these findings may be that T cells at sites of pathology may maintain a distinct metabolism from those in circulation. Alternatively, the metabolic signatures of immune cells generated *in vitro* versus *in vivo* may be fundamentally different, and investigating the similarities and differences between these cells could reveal aspects of the metabolism–inflammation relationship that are currently being overlooked (97). The study of Treg metabolism provides a great example of the discrepancies between *in vivo* and *in vitro*-derived cells. Whereas Tregs (Tregs) generated by standard *in vitro* protocols maintain a metabolic profile that favors mitochondrial respiration over aerobic glycolysis, Tregs isolated *ex vivo* seem to be profoundly glycolytic (51, 98), and this metabolic signature is proposed to favor their transcriptional activity at *Eno1* to produce α-enolase and MBP-1 (43). Indeed, the association between glycolytic flux and inflammation is likely not as clear *in vivo* as it is *in vitro*. Nevertheless, the non-metabolic functions of glycolytic machinery, including their relationship to inflammation, have been convincingly demonstrated *in vivo* and represent intriguing therapeutic opportunities for the future development of metabolically focused interventions for inflammatory disease. Toward this end, it will be important to determine the non-metabolic functions of glycolytic enzymes in other systems. For example, the mitochondrial localization of HK-II is important for cardiomyocyte function and interventions at this level of glycolysis might be anticipated to impact the heart (15, 16). In the kidney, podocytes were recently shown to express high levels of PKM2, and the non-metabolic functions of the enzyme in this context appear to play essential disease-potentiating roles in the context of diabetic nephropathy (77). Indeed, an important step toward realizing the therapeutic potential of targeting the non-metabolic functions of glycolytic enzymes during inflammation is to better understand these functions within and beyond the context of immunity. Finally, just as particular inflammatory processes are often associated with a unique cytokine profile (e.g., TNF-α and rheumatoid arthritis or IL-17A and psoriasis), the metabolic proclivities and peculiarities of cells driving inflammation may also differ based on disease-specific contexts. Further investigation into the nuances of immune cell metabolism in the *in vivo* setting and how this relates to their inflammatory functions are needed to better elucidate and potentially target the relationship between metabolism and inflammation during disease.

## Conclusion

The metabolic requirements that support immune-mediated inflammatory responses are well established *in vitro* and increasingly so *in vivo*. Elevated consumption of glucose plays an important role in inflammatory responses of T cells, where glycolytic processes can serve to generate ATP, produce metabolic intermediates that are important for anabolic processes and even alter the epigenetic landscape of the activated cell. To achieve this, activated immune cells must upregulate expression of metabolic machinery, many of which serve non-metabolic functions in the cell that are directly linked to modulating the inflammatory response. Research in cancer cells has led to the identification of many non-metabolic functions of glycolytic enzymes (100, 101), and only recently are these functions beginning to be assessed in the context of inflammation. Just as research into the metabolic activity of cancer cells provided the foundations for immunometabolic studies to identify the unique bioenergetic requirements of immune cell subsets, so too may the non-metabolic functions of glycolytic enzymes discovered in cancer cells instruct an alternative way of looking at the relationship between metabolism and inflammation. Importantly, this alternative approach may generate interventions that are more readily translatable to the clinical setting than therapies that overtly impinge on enzymatic activity of metabolic machinery.

In addition to those listed here, other isoforms of glycolytic machinery with known non-metabolic properties in cancer cells, such as phosphofructokinase-1 (102), seem to be selectively induced in immune cells in response to distinct stimuli. Determining how these contribute to the T cell inflammatory program is of interest. Conversely, activation-induced proteins that are not classically associated with metabolism, such as CD69 (103), may also play metabolic roles that are important for inflammatory immune responses. In addition, byproducts of metabolic processes, such as PEP (10), lactate (104, 105), succinate (19, 66, 106–108), citrate (109), 2-hydroxyglutarate (110), α-ketoglutarate (111), and others (102), are gaining increasing recognition for the non-metabolic roles they play as direct modulators of inflammation. Further exploration into the unique ways in which metabolic processes contribute to immune responses may reveal exploitable opportunities to destabilize the relationship between metabolism and inflammation for therapeutic benefit.

## Author Contributions

Both the authors have made a substantial, direct, and intellectual contribution to the work and approved it for publication.

## Conflict of Interest Statement

The authors declare that the research was conducted in the absence of any commercial or financial relationships that could be construed as a potential conflict of interest.
